# Effect of Additive Friction Stir Deposition Processing on the Microstructure and Mechanical Properties of 1045 Steel

**DOI:** 10.3390/ma18061257

**Published:** 2025-03-12

**Authors:** Wei Lei, Xudong Ran, Qi Wang, Yang Wu, Jipeng Sun, Feiyue Zhang, Shuhai Huang, Lin Xiang, Jianquan Tao, Qiang Chen

**Affiliations:** 1 Southwest Technology and Engineering Research Institute, Chongqing 400039, China; autismpait@gmail.com (W.L.); xudongran0828@outlook.com (X.R.); bruce_xlin@163.com (Q.W.); cquwuyang@163.com (Y.W.); sun1554384191@163.com (J.S.); 18723003055@163.com (F.Z.); hsh82@163.com (S.H.); xlin0731@163.com (L.X.); 2 School of Mechatronics Engineering, Harbin Institute of Technology, Harbin 150001, China

**Keywords:** 1045 steel, additive friction stir deposition, microstructure, mechanical properties, strengthening mechanism

## Abstract

Using additive friction stir deposition (AFSD), the poor weldability of 1045 steel can be solved, facilitating the efficient and high-performance additive manufacturing of its components. This study selected spherical 1045 steel powder and investigated key factors influencing mechanical properties, including deposition temperature, tool rotational rate, and axial force. The results showed that dynamic recrystallization (DRX) occurred in AFSD 1045 steel, which produced randomly oriented fine equiaxed grains with a size range of 1–3 µm and was sensitive to changes in tool rotational rate and axial force. The AFSD 1045 steel, with a maximum surface hardness of 477.2 HV, ultimate tensile strength of 1061.9–1172.3 MPa, and elongation of 8.6–19.0%, has superior overall mechanical properties compared with other forming processes. Moreover, by analyzing tensile fracture morphology, geometrically necessary dislocation (GND) density, and coincidence site lattice (CSL) boundary distribution characteristics, the strengthening mechanism in AFSD 1045 steel was discussed. The research findings serve as a reference for optimizing the AFSD process for 1045 steel and supply a new alternative for joining and manufacturing this material.

## 1. Introduction

Traditional subtractive manufacturing (SM), which includes processes such as drilling, turning, milling, and boring, typically generates considerable raw material waste [1,2,3]. Moreover, as metal parts become increasingly complex, integrated, and larger, their manufacturing becomes more costly and challenging. In contrast, additive manufacturing (AM) employs a discrete-stacking approach that utilizes metallic powders, wires, or rods as raw materials. The layer-by-layer construction of components is achieved under computer numerical control (CNC) through high-energy heat sources such as lasers, electron beams, and plasma arcs, or solid-state methods like sheet lamination and additive friction stir deposition (AFSD) [4]. Consequently, AM presents a faster and more cost-effective method for producing complex components compared to SM.

In recent decades, numerous metallic AM technologies have emerged, including cold spraying (CS) [5], ultrasonic additive manufacturing (UAM) [6], laser powder bed fusion (L-PBF) [7], direct energy deposition (DED) [8], etc. However, specific bottlenecks still exist in current AM techniques. Solid-state AM technologies, such as CS-AM and UAM, are prepared metal materials that exhibit considerable variation in their mechanical properties, influenced by factors involving lattice strain, non-bonding zones, and changes in grain size [9]. These materials often display intrinsic cracks, poor continuity, and limited plasticity [10,11]. Liquid-state AM technologies, exemplified by L-PBF and DED, also suffer from casting defects [12,13], inhomogeneous material properties [14], and shortcomings in processing efficiency.

AFSD, based on stir friction welding, overcomes the limitations of both solid-state and liquid-state AM processes, garnering considerable attention in the application of aluminum alloys. For example, Perry et al. investigated the interface characteristics after AFSD between Al-Mg-Si alloys (6061 aluminum alloy) and Al-Cu alloys (2024 aluminum alloy), finding that metallurgical bonding occurs between the two dissimilar aluminum alloys and the deposited layer of the 2024 aluminum alloy [15]. Rivera et al.’s research indicates that dynamic recrystallization occurs in AFSD materials, resulting in a microstructure resembling that of wrought materials [16,17], thereby yielding relatively superior mechanical properties in the fabricated metallic materials. Despite this, there are few reports on AFSD-related studies concerning steel materials. 1045 steel is a widely available engineering material, with relatively poor weldability. Given the specific advantage of AFSD for weakly weldable materials [18,19,20], there are few reports on AFSD-related studies concerning steel materials. Therefore, the objective of this study is to demonstrate the possibility of preparing 1045 steel material by AFSD, and to investigate the effects of the AFSD rotational speed and force product (the product of tool rotational rate and axial force, PoRF) and deposition temperature on the microstructure and mechanical properties of 1045 steel, providing references to promote the application of AFSD.

## 2. Materials and Methods

**Figure 1:**
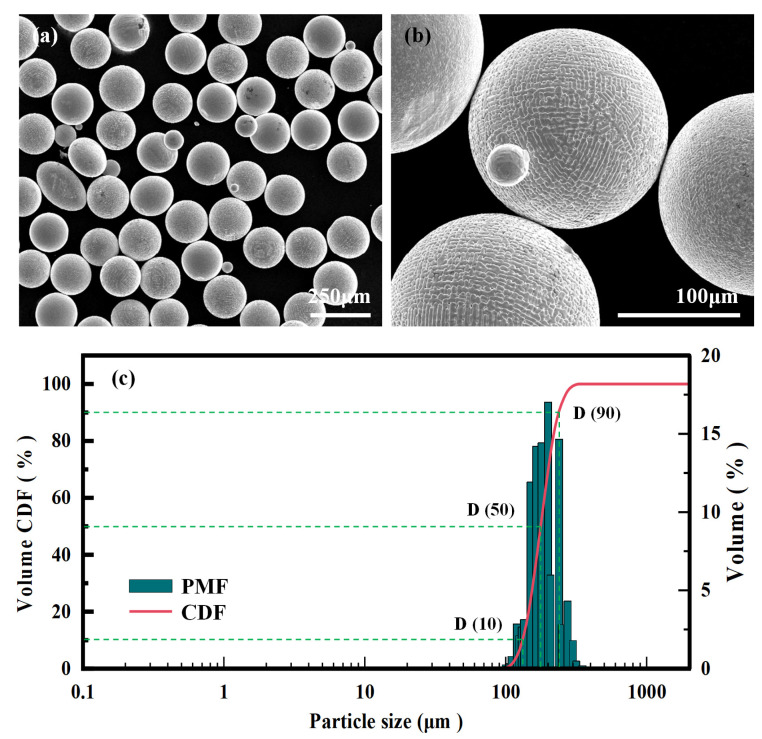
1045 steel powder: (**a**) 200× and (**b**) 1000× SEM, (**c**) particle size distribution.

Rolling-annealed 1045 steel, measuring 400 × 400 × 25 mm, is selected as the substrate for the AFSD process. The raw material consists of spherical 1045 steel powder, prepared using the plasma-rotating electrode process, with its chemical composition detailed in Table 1. Scanning electron microscope (SEM, Hitachi S-4800, Tokyo, Japan) images of the powder are presented in Figure 1a,b. As shown in Figure 1a, it can be observed that the size distribution of the 1045 steel powder is uniform and exhibits good sphericity, with no broken or hollow particles. However, a minor amount of satellite and non-spherical powder is also present. A further examination of Figure 1b reveals that the powder’s surface is smooth and features a columnar-type crystal structure caused by rapid solidification following melting. Figure 1c shows the particle size distribution of 1045 steel powder. The distribution is notably concentrated, with particle sizes ranging from 150.0 to 239.0 μm, accounting for 24.2% to 90.4% of the cumulative volume distribution of the powder.

**Figure 2:**
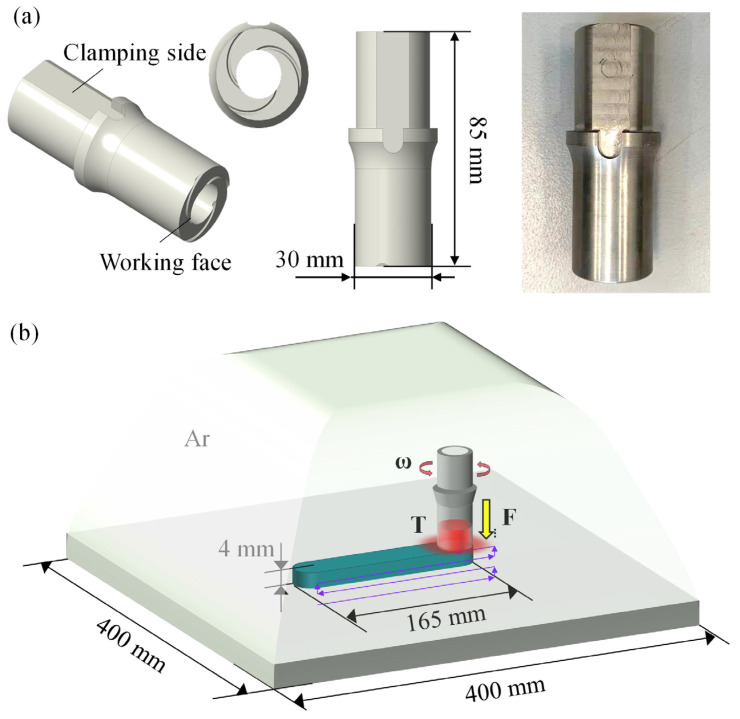
AFSD experiment: (**a**) tool and (**b**) schematic diagram of deposition sample.

Figure 2 presents a schematic diagram of the AFSD experiment. To optimize deposition quality while controlling costs, a tool with a shoulder diameter of 30 mm was utilized. The working face of the tool was designed in a spiral structure, which facilitates the aggregation of powder during rotation (Figure 2a). The experiment was conducted in a pure argon atmosphere (99.99%). Initially, the tool was rotated to the target rotational speed, after which an auxiliary heating module was activated to heat the powder. The tool was then lowered until it contacted the substrate and reached the target axial force, at which point the deposition temperature was recorded. The tool moved at 80 mm/min, covering a deposition layer of 165 mm in the *X*-axis direction. Following this, the tool was raised by 0.8 mm in the *Z*-axis direction and then returned to the 165 mm position in the *X*-axis direction, resulting in a total thickness of 4 mm for the AFSD specimen (Figure 2b).

As previous studies have suggested, the deposition temperature (T), tool rotational speed (ω), and axial force (F) are critical parameters in AFSD-deposited materials that affect their microstructure and mechanical properties. When the T is below 700 °C, the internal metallurgical bonding of the deposited material remains incomplete. Conversely, T exceeding 900 °C leads to the material adhering easily to the inner wall of the tool. The suitable range for ω is 300–600 rpm. At lower ω, the grain size uniformity of the deposited material is compromised, while higher ω enhances the plastic flow of materials, which can negatively impact deposition quality. The optimal value for F is between 6 kN and 12 kN. Lower values may result in porosity at the particle interfaces, reducing the density of the deposited material, while higher values can accelerate tool head abrasion. In light of these findings, experiments were conducted using AFSD with varying process parameters, as detailed in Table 2.

**Figure 3:**
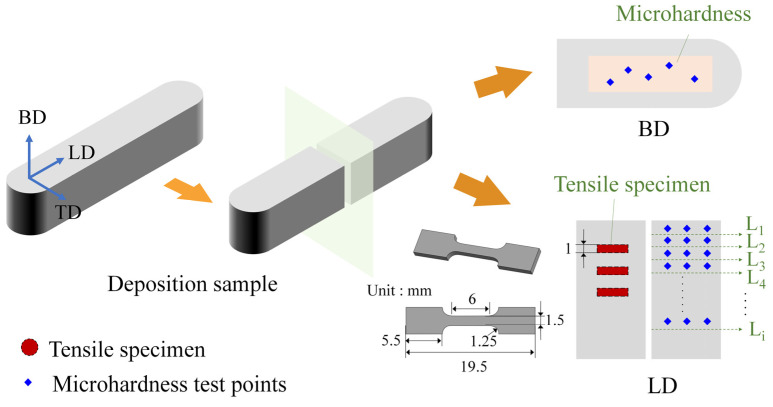
Schematic diagram of hardness test points and tensile specimen sampling positions.

The temperature of the deposited materials was monitored using a two-color pyrometer (STRONG-GR-3514, Sijie, Changzhou, China). The specimens for observation were cut from the upper surfaces of deposited specimens along the LD-TD section, which were mechanically polished and etched with an electrolyte containing 4% nitric acid and 96% alcohol for 15 s at room temperature. An optical microscope (OM, Olympus PME-3, Tokyo, Japan) and a scanning electron microscope (SEM, Jeol JSM 7200F, Tokyo, Japan), equipped with an electron backscatter diffraction probe (EBSD, Ametek EDAX Velocity Super, CA, USA), were employed to observe the microstructure. Subsequently, the microstructure images were processed using ImageJ 1.53 (NIH, Bethesda, MD, USA) and Channel 5 (HKL, Eselsberg, Ulm, Germany) software. The mechanical properties of the AFSD specimens were evaluated using a microhardness tester (AHVD-1000, Jujing, Shanghai, China) and an electronic universal material testing system (INSTRON 5587, Boston, MA, USA). Figure 3 illustrates the measurement points used for microhardness and tensile testing. The surface hardness of the sample was obtained by averaging the results of five randomly performed tests. For cross-section hardness, three sequential measurements were taken along the depth range from L_1_ to L_i_, applying a load of 1 kgf (9.807 N) for 15 s. To minimize error, the tensile tests were conducted at least in triplicate along the LD direction, with the strain rate at 0.1 mm/min and at room temperature.

## 3. Results and Discussion

### 3.1. Microstructure Analysis

#### 3.1.1. Grain Size

**Figure 4:**
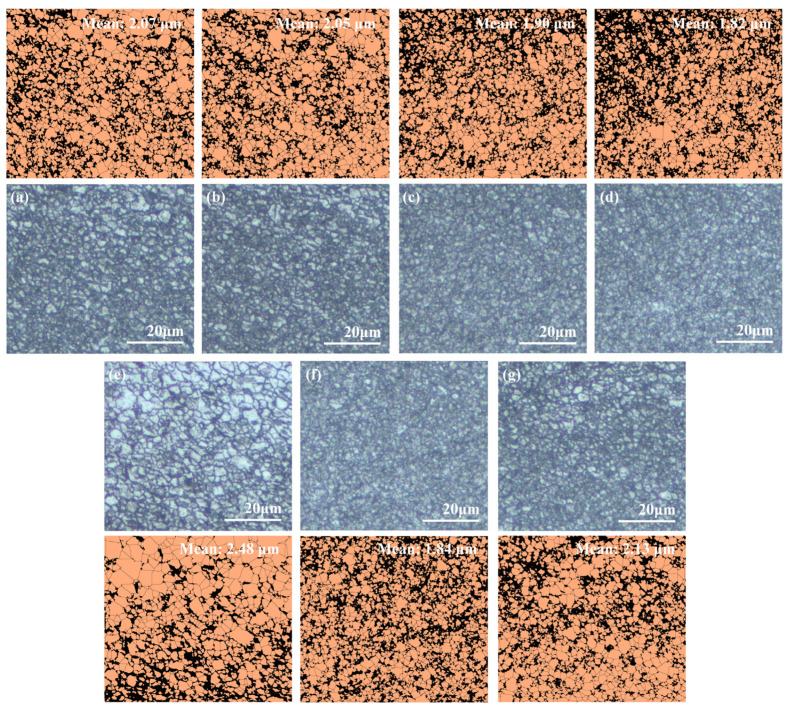
OM images of AFSD specimens with varying parameters, along with ImageJ-processed grain statistics plots: (**a**) No. 1, (**b**) No. 2, (**c**) No. 3, (**d**) No. 4, (**e**) No. 5, (**f**) No. 6, and (**g**) No. 7.

Metallographic images of specimens numbered 1 to 7 in Table 2 were obtained using OM, as illustrated in Figure 4a–g. These images were processed using ImageJ to conduct a statistical analysis of the average grain sizes. The 1045 steel materials deposited by the AFSD exhibit fine grains with an equiaxed crystal structure, with an average grain size of less than 3 μm.

**Figure 5:**
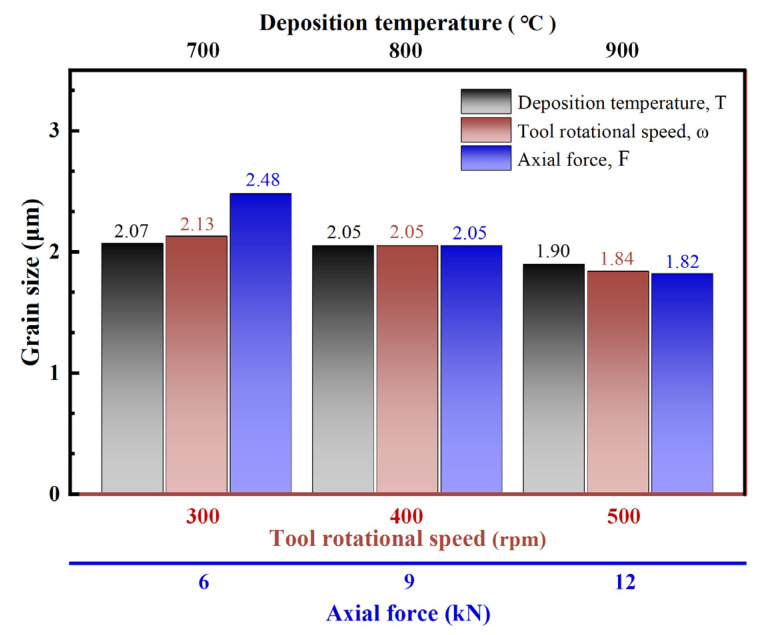
Variation in AFSD 1045 steel sample grain size with T, ω, and F.

From Figure 5, it is evident that the grain sizes of AFSD specimens exhibit a decreasing trend with increasing T, ω, and F. When T increases from 700 °C to 900 °C, the grain size of the specimens decreases from 2.07 to 1.90 μm, representing a reduction of 8.2% (Figure 4a–c). For ω, when it increases from 300 to 500 rpm, the specimen grain size decreases from 2.13 to 1.84 μm, resulting in a reduction of 13.6% (Figure 4b,f,g). When F increases from 9 to 12 kN, the specimen grain size decreases from 2.48 to 1.84 μm, corresponding to a reduction of 26.8% (Figure 4b,d,e). These results demonstrate that variations in the AFSD process parameters affect the grain size of the deposited specimens, with greater sensitivity observed in response to changes in ω and F. During the AFSD process, F and ω exert compressive and shear forces on the raw materials, keeping them in a state of high strain and strain rate [21,22], which induces intense plastic deformation. Therefore, to assess the severity of raw material deformation during the AFSD process, we consider the product of rotational speed and force (**PoRF**), defined as the product of ω and F, as referenced in Equation (1).

(1) PoRF=ω·F

where ***ω*** is the tool rotational speed (rpm) and ***F*** is the axial force (kN).

**Figure 6:**
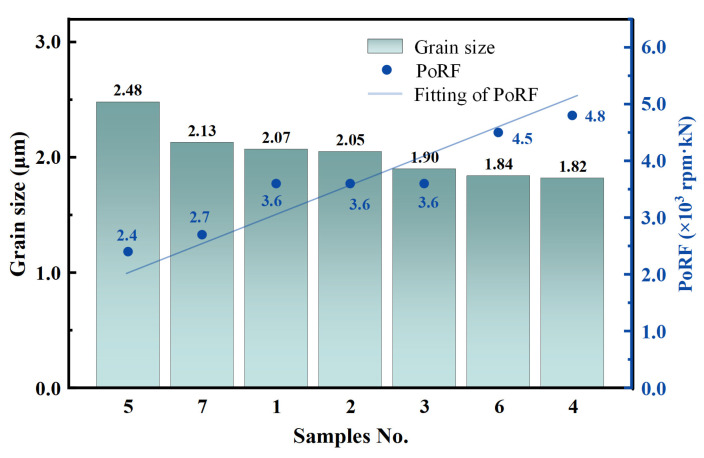
Grain size and PoRF of different AFSD 1045 steel samples.

Figure 6 depicts the grain size (histogram) and PoRF (scatter plot) of different AFSD specimens, sorted in order of grain size from largest to smallest. As shown in the plots and Table S1, the grain size of AFSD specimens is negatively correlated with PoRF. Specimen 4 (No. 4) exhibits the smallest grain size and the largest PoRF among all specimens (grain size of 1.82 μm, PoRF of 4.8 × 10^3^ rpm·kN), while specimen 5 (No. 5) has the largest grain size and the smallest PoRF (grain size of 2.48 μm, PoRF of 2.4 × 10^3^ rpm·kN). This indicates that when T is above 700 °C, the effect of temperature on the grain refinement of 1045 steel AFSD specimens is not significant, while the high strain rate resulting from the high rotational speed of the tool and the large deformation caused by the axial force are more likely in causing the grain refinement.

#### 3.1.2. Recrystallization Characteristics

**Figure 7:**
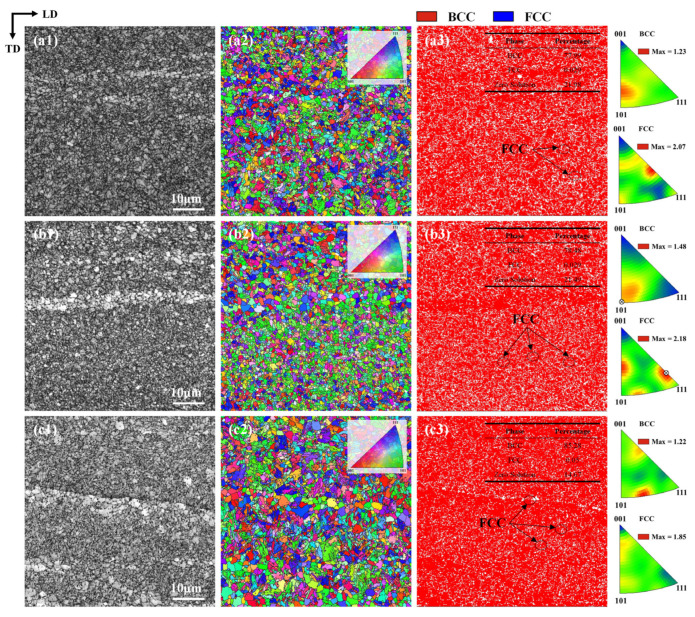
The BC maps, IPF-Z maps, and phase distribution maps together with IPF-Z triangle plots of the deposited specimens: (**a1**–**a3**) No. 1, (**b1**–**b3**) No. 2, (**c1**–**c3**) No. 4.

The processing state of AFSD 1045 steel can be equated to hot working combined with severe plastic deformation, which is typically accompanied by phenomena such as recovery, recrystallization (DRX), and texturing. The EBSD specimens cut from the upper surfaces of deposited specimens 1, 2, and 4 were scanned along the BD direction after grinding and electrolytic polishing to investigate the effects of process parameters on the evolution of the specimen microstructure. Among these, No. 1 and No. 2 have the same PoRF but different T, while No. 2 and No. 4 have the same T but different PoRF. The band contrast (BC) map (Figure 7(a1–c1)) and inverse pole figure-Z (IPF-Z) coloring map (Figure 7(a2–c2)) reveal that the deposited specimens contain a significant number of randomly oriented fine equiaxed grains, with an average grain size ranging from about 1 to 3 μm. Additionally, a large area of low contrast in the BC map indicates substantial strain within the specimens. Figure 7(a3–c3) present the phase distribution and IPF triangle plots of the deposited samples. The body-centered cubic (BCC) ferrite phase (α-Fe) and a small amount of face-centered cubic (FCC) austenite phase (γ-Fe) precipitated from grain boundaries are observed in the AFSD specimens. As the T increases, the driving force for austenitizing is enhanced, resulting in a higher α-Fe content (No. 1 and No. 2). Moreover, when PoRF increases, severe plastic deformation disrupts the primitive grains, providing numerous nucleation sites for γ-Fe, which increases its content in No. 2 and No. 3. Specifically, the γ-Fe content of No. 1 and No. 2 is 0.029%, while the γ-Fe content of No. 4 is 0.05%. From the α-Fe and γ-Fe phase IPF-Z triangular plots (CS0 coordinate system), it is evident that the deposited specimens do not exhibit apparent texture. No. 2 shows a weakly preferred orientation in the Z direction, with α-Fe high-density poles concentrated in the {110} direction, exhibiting a polar density maximum of 1.48, while γ-Fe density poles are concentrated in the {223} direction, with a polar density maximum of 2.18.

**Figure 8:**
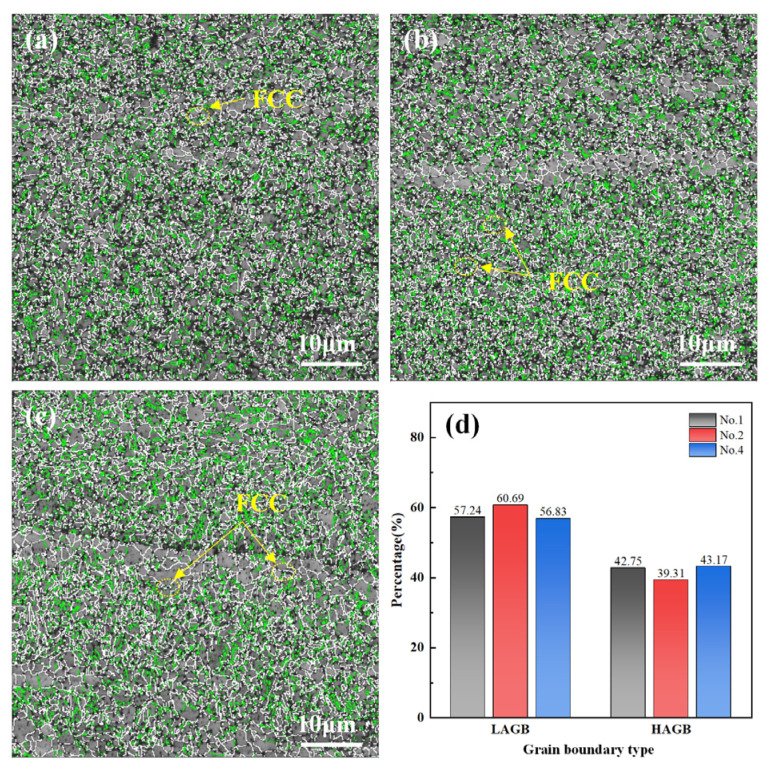
The grain boundary characteristics of the AFSD samples: (**a**) No. 1, (**b**) No. 2, (**c**) No. 4, and (**d**) the percentage of each grain boundary type.

Figure 8a–c illustrate the grain boundary distribution characteristics of specimens 1, 2, and 4. The white and green curves represent high-angle grain boundaries (HAGBs, >15°) and low-angle grain boundaries (LAGBs, 2°–15°). As shown in Figure 8a–c, increasing T raises the percentage of LAGBs in No. 1 and No. 2 from 57.24% to 60.69%. However, a higher PoRF reduces the percentage of LAGBs in No. 4 to 56.83%. Increasing T exacerbates atomic vibrations, making dislocations more prone to slip and accumulate in areas of severe deformation, thus forming LAGBs. In contrast, increasing PoRF enhances lattice distortion, allowing some dislocations to bypass LAGBs and rearrange, resulting in fewer LAGBs [23]. Additionally, raising T drives nucleation, further increasing LAGB content, while increasing PoRF elevates stored deformation energy and promotes the DRX of deformed grains, leading to a reduction in LAGB content.

**Figure 9:**
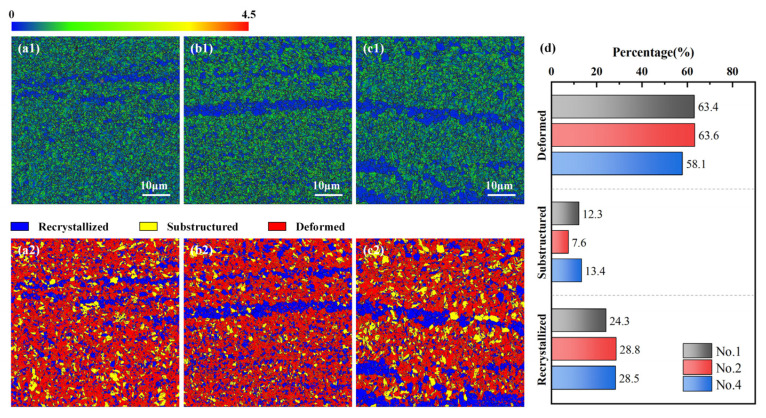
KAM Maps and distribution characteristics of dynamically recrystallized (DRX) grains in the deposited samples: (**a1**,**a2**) No. 1, (**b1**,**b2**) No. 2, (**c1**,**c2**) No. 4, and (**d**) the percentage of each grain type.

Figure 9(a1–c1) shows the KAM map of the deposited samples, with a relatively uniform distribution of local misorientation for samples 1, 2, and 4. In comparison to Figure 8a–c, it is noticed that the local misorientation is primarily concentrated at the LAGBs or subgrain boundaries formed by the annihilation and rearrangement of dislocations, along with significant local misorientation around γ-Fe. During the AFSD process, the deposited material accumulates deformation energy through cyclic severe deformation, which deforms the grains, increases defect density, and generates a substantial fraction of dislocations. The proportions of deformed grains in specimens 1, 2, and 4 are 63.4%, 63.6%, and 58.1%. Furthermore, the temperature of the deposited material (50% to 90% Tm) consistently exceeds the DRX temperature [24], enabling continuous recovery and DRX, as indicated by the yellow and blue grains in Figure 9(a2–c2). The recovery process occurs without grain boundary migration, generating substructures that retain high stresses and local misorientation, with percentages of substructure in specimens 1, 2, and 4 being 12.3%, 7.6%, and 13.4%, respectively. Following the DRX process in AFSD, dislocations are in a dynamic state of accumulation, slippage, annihilation, and regeneration. Regions with high local misorientation lead to dislocation accumulation, producing crystal nuclei with lower local misorientation that gradually form DRX grains. The percentages of DRX grains for No. 1, No. 2, and No. 4 are 24.3%, 28.8%, and 28.5%.

### 3.2. Mechanical Properties

#### 3.2.1. Vickers Hardness

**Figure 10:**
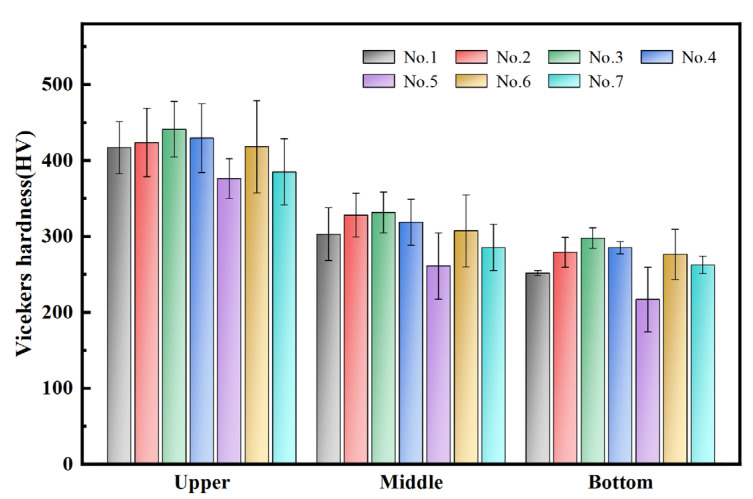
Hardness of different areas of the 1045 steel deposited sample.

The BD-TD cross-section of the 1045 steel deposited specimen was divided into three sections: upper (0 to 1000 μm), middle (1000 to 3000 μm), and bottom (3000 to 4000 μm). Hardness testing was conducted by sequentially taking measurements at 400 μm intervals along the BD direction from the surface of the specimen. The results of the hardness statistics for the specimens under different process parameters are presented in Figure 10 and Table S2. The mean and standard deviation of hardness exhibit a decreasing trend from the upper to the bottom sections, with greater fluctuations in hardness observed in the upper section compared to the bottom. This indicates that the hardness in the upper section is more sensitive to variations in process parameters. The average hardness across different areas of each sample cross-section was calculated, revealing the following order of hardness from highest to lowest: 3 > 2 > 4 > 6 > 1 > 7 > 5.

**Figure 11:**
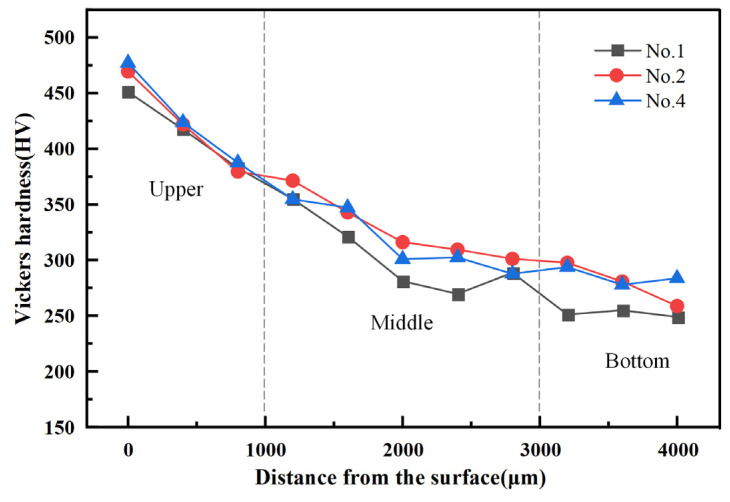
Hardness distribution of samples 1, 2, and 4 along the BD direction.

Figure 11 illustrates the hardness distribution along the BD direction for specimens 1, 2, and 4, highlighting a decreasing trend in hardness from the upper to the bottom sections. The average hardness of specimens 1, 2, and 4 are 320.2 HV, 340.8 HV, and 339.7 HV, respectively. Notably, the highest hardness recorded on the surface of the deposited specimen is 477.2 HV (No. 4), while the highest hardness at the bottom is 340.8 HV (No. 2). This phenomenon may be attributed to the fact that, compared to the surface of the deposited specimen, the bottom material experienced the cumulative effects of multiple thermal cycles during the AFSD process. Consequently, the bottom material effectively underwent a longer annealing treatment, resulting in a reduction in dislocation density within the deformed grains, the elimination of substructures such as entangled dislocations, and grain growth, which collectively led to a decrease in hardness.

#### 3.2.2. Tensile Properties

**Figure 12:**
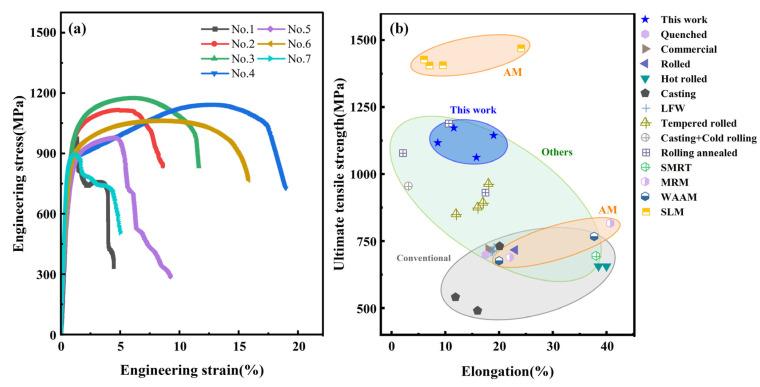
Tensile properties of the AFSD 1045 steel samples: (**a**) engineering stress–strain curve, (**b**) comparison with other forming methods reported in the publication, containing quenched [25], linear friction welded (LFW) [25], tempered rolled [26], commercial [27], selective laser melting (SLM) [27,28], rolled [28], hot rolled [29], surface mechanical rolling treatment (SMRT) [29], casting [30,31], casting + cold rolling [30], rolling annealed [30], metamorphic rolling mechanism (MRM) [31], and wire arc additive manufacturing (WAAM) [31].

Figure 12a presents the engineering stress–strain curves of the 1045 steel deposited specimens prepared with different AFSD process parameters. The specific values of mechanical properties, such as yield strength (YS), ultimate tensile strength (UTS), and elongation of the specimens, are provided in Table 3. By comparison, the mechanical properties of the specimens are positively correlated with T and PoRF within the parameter range of this research. When comparing No. 1 and No. 3 (with T of 700 °C and 900 °C, respectively), the YS and UTS increased by 23.8 MPa and 195.6 MPa, while elongation increased from 4.4% to 11.6%. In comparing No. 7, No. 6, and No. 5, No. 4 (where the former’s ω was increased by 200 rpm and the latter’s F was increased by 6 kN), the UTS of No. 6 and No. 4 both increased by 168.0 MPa, and the elongation increased by 12.3% and 12.7%, respectively. Additionally, all specimens except No. 1 and No. 7 exhibited some degree of work hardening. Figure 12b compares the UTS and elongation of 1045 steel materials produced in this study with those from other forming processes [25,26,27,28,29,30,31]. As shown in Figure 12b, most of the forming methods reported in the publications for 1045 steel materials, such as quenching, rolling, and wire arc additive manufacturing (WAAM), struggle to achieve both high UTS and elongation. However, the AFSD process used in this study better balances the strength and plasticity of 1045 steel, achieving a UTS 1061.9–1172.3 MPa and elongation 8.6–19.0%.

**Figure 13:**
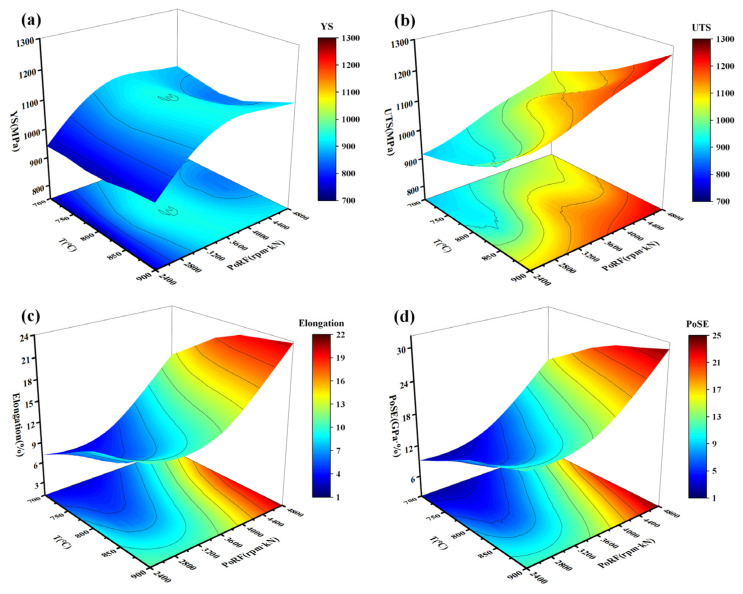
Correlation between process parameters and mechanical properties: (**a**) yield strength (YS), (**b**) ultimate tensile strength (UTS), (**c**) elongation, and (**d**) product of strength and elongation (PoSE).

The effects of the AFSD process parameters on the mechanical properties of the deposited samples are illustrated in Figure 13, where a deeper red color indicates a higher value of the corresponding mechanical property. The results demonstrate that the T and PoRF significantly influence the UTS and elongation of the samples (Figure 13b,c), while the YS shows insensitivity to changes in both parameters (Figure 13a). In practical processing parameter optimization, it is often necessary to consider both strength and plasticity. Therefore, the product of strength and elongation (PoSE, Table 3) is employed to assess the overall mechanical properties of AFSD materials. From Figure 13d, it is evident that within the process window of T 850–900 °C and PoRF 4400–4800 rpm·kN, the AFSD 1045 steel material can attain a relatively favorable strength–plasticity matching.

### 3.3. Strengthening Mechanism

**Figure 14:**
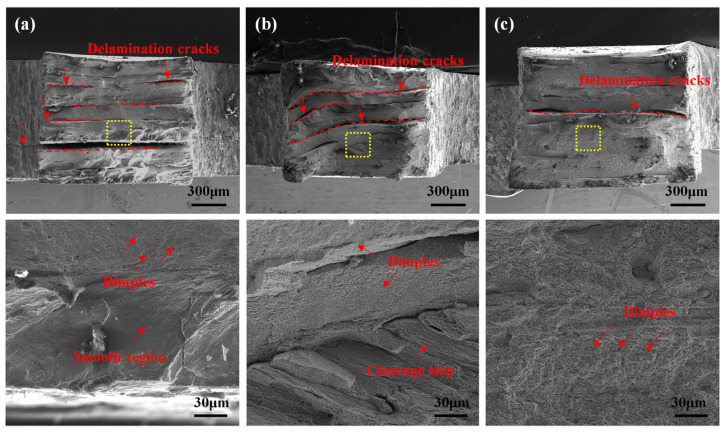
Morphology of fracture in AFSD tensile specimens: (**a**) No. 1, (**b**) No. 2, and (**c**) No. 4.

To further understand the fracture mechanism of the AFSD 1045 steel material, the fracture morphology of the tensile specimens was analyzed. Figure 14 illustrates the low and high-magnification fracture morphologies of the deposited specimens 1, 2, and 4 after the tensile test. In Figure 14a, observed at high magnification, a large number of fine pore-like dimples and some smooth regions indicate poor plasticity. The smooth regions, caused by friction and sliding on the fracture surface, typically represent brittle fractures [32]. Figure 14b shows cleavage steps and fine dimples in the fracture, suggesting a transition in the fracture mode from a brittle fracture to a mixed mode of cleavage and ductile fracture. In Figure 14c, only dimple features are observed, with significantly larger dimples indicating that No. 4 exhibits good plasticity and is characterized by ductile fracture. Notably, delamination bifurcation cracks were observed in the low magnification fractures of specimens 1, 2, and 4. These may result from uneven strain and stress distribution within the deformed grains. Typically, the formation of a moderate amount of delamination cracks facilitates the dissipation of localized stress concentrations in the material, resulting in a three-dimensional stress shift at the crack tip [33,34]. The pile-up dislocations within the deformed grains and at the grain boundaries of the deposited material can reduce the elastic stress field at the crack tip, producing a shielding effect [35,36,37]. Additionally, precipitated heterogeneous particle phases, such as carbides and silica [27,38], can deflect the crack propagation path [39,40], thereby improving the plasticity of the material.

**Figure 15:**
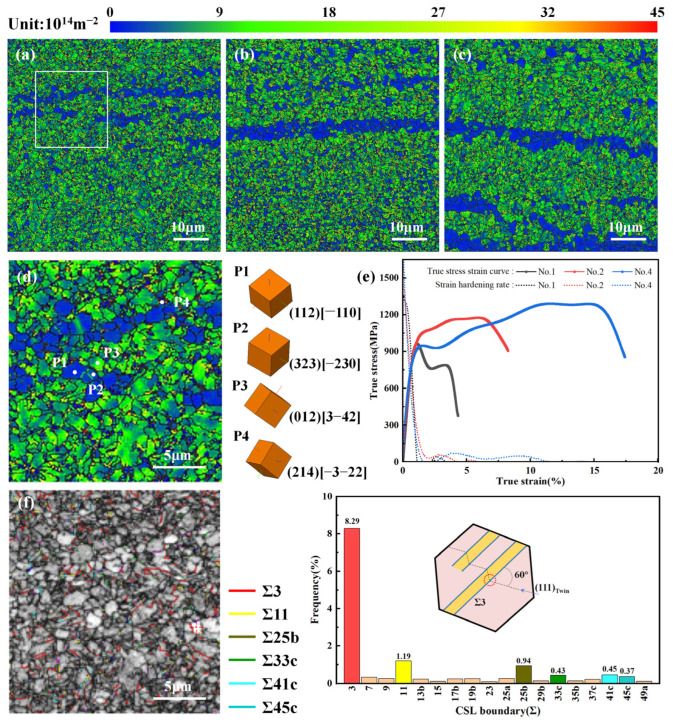
Dislocation strengthening and twinning strengthening in 1045 steel deposited specimens: (**a**–**c**) GND density map of specimens 1, 2, and 4, (**d**) grain orientation characteristics at typical locations of No. 1, (**e**) true stress–strain and work-hardening curves of specimens 1, 2, and 4, (**f**) CSL boundary distribution characteristics of No. 1.

Figure 15a–c shows the GND density maps of samples 1, 2, and 4. It is observed that the GND density is high within the material, with an average value of approximately 2 × 10^14^–3 × 10^14^ m^−2^. Additionally, the GND density is not uniformly distributed, and it is primarily concentrated in the substructures and deformed grains (Figure 9). Figure 15d displays the grain orientation at the typical position of No. 1 and calculates the GND density, revealing that the GND density in different regions varies by nearly an order of magnitude. Specifically, the GND densities of recrystallized (P1), substructure (P2), and deformed grains (P3) are 2.61 × 10^14^  m^−2^, 3.98 × 10^14^  m^−2^, and 14.48 × 10^14^  m^−2^, while the GND density at grain boundaries (P4) reaches 40.74 × 10^14^ m^−2^, which is 15.6 times higher than the recrystallized grains. Meanwhile, the significant accumulation of dislocations within the specimen leads to entanglement, creating pinning points that hinder dislocation movement and consequently contribute to work hardening (Figure 15e). Figure 15f illustrates the distribution characteristics of coincidence site lattice (CSL) boundaries in the white region of Figure 15a, revealing a high percentage (8.29%) of sigma (Σ) 3 boundaries. The Σ3 boundary is a type of twin boundary with the highest interface overlap among all low Σ CSL boundaries, exhibiting low-energy characteristics. Co-lattice Σ3 grain boundaries dissipate the energy stored in the material, increase the resistance to crack propagation, and enhance the plasticity of the material [41,42], as well as provide nucleation sites for dislocations and impede dislocation motion, thereby increasing the strength of the material [43]. In general, Σ3 grain boundaries tend to appear in FCC metals, while BCC metals with high stacking fault energy lack intrinsic stacking faults. Consequently, they must overcome ultra-high potential barriers for twin nucleation [44], which typically occurs under extreme conditions such as low temperature, high strain rate, and rapid cooling [28,45,46]. Moreover, Li et al. [28] reported the presence of distinct twinning interfaces in 1045 steel prepared by selective laser melting (SLM). Therefore, the large number of Σ3 (111) 60° interfaces in No. 1 suggests that twins may have formed in the AFSD specimen.

According to the aforementioned study, the strengthening mechanisms of 1045 steel produced by the AFSD process can be classified as (i) grain boundary strengthening (***σ_g_***), (ii) dislocation strengthening (***σ_d_***), (iii) twin boundary strengthening (***σ_t_***), (iv) solid solution strengthening (***σ_s_***), and (v) precipitation strengthening (***σ_p_***). The total strengthening effect (Δ***σ***) is shown in Equation (2).

(2) $$∆\sigma \;={\;\sigma }_{0}+\sigma_{g}+\sigma_{d}+\sigma_{t}+\sigma_{s}+\sigma_{p}$$

where ***σ***_0_ denotes the lattice friction, which can be calculated by the Peierls–Nabarro formula (Equation (3)).

(3) $${\;\sigma }_{0}=G\cdot \exp(-\frac{2\pi w}{b})$$

In this context, ***G*** represents the shear modulus (8.3 × 10^4^ MPa), ***W*** denotes the dislocation width, ***b*** signifies the Burgers vector (0.248 nm), and ***σ***_0_ is estimated to be 48 MPa.

The process of grain refinement in metallic materials leads to an increase in the proportion of grain boundaries, which impedes the movement of dislocations and induces a strengthening effect. The relationship between grain size and the strengthening effect can be expressed by the Hall–Petch formula (Equation (4)).

(4) $$\sigma_{g}=K_{y}d^{-0.5}$$

where ***K_y_*** is the Hall–Petch constant (13.4 MPa·mm^1/2^) of the substrate [47] and ***d*** is the average grain size of the deposited material.

The dislocation reinforcement was estimated by the Bailey–Hirsch formula (Equation (5)).

(5) $$\sigma_{d}=\mathrm{MaGb}\rho^{0.5}$$

where ***M*** represents the Taylor factor (3.067), ***a*** is a constant related to the crystal structure (0.3), and ***ρ*** is the dislocation density, defined as the sum of the geometrically necessary dislocation (GND) density and the statistically stored dislocation (SSD) density (Equation (6)).

(6) $$\rho =\rho_{\mathrm{GND}}+\rho_{\mathrm{SSD}}$$

The calculated ***ρ_GND_*** obtained using the Hough-based EBSD method is 2 × 10^14^–3 × 10^14^ m^−2^. Challenges remain in accurately measuring ***ρ_SSD_*** [48], but the use of diffraction line profile analysis (DLPA) can provide detailed information about dislocations [49].

Twin boundary strengthening is a type of grain boundary strengthening that satisfies the Hall–Petch formula and can be calculated using Equation (7) [50].

(7) $$\sigma_{t}=FK_{t}S^{-0.5}$$

where ***F*** is the volume fraction of twins (%), ***K_t_*** is the Hall–Petch constant of the twin boundary, which is usually smaller than the matrix and is taken as 13.4 MPa·mm^1/2^, and ***S*** is the twin spacing, which is calculated by Equation (8).

(8) $$\frac{1}{S}=\frac{1}{2e}\cdot \frac{F}{1-F}$$

In this context, ***e*** represents the twin thickness.

Different solute atoms can cause lattice distortion, hindering dislocation motion and thereby achieving a strengthening effect. Solid solution strengthening can be calculated by Equation (9) [51].

(9) $$\sigma_{s}=M\cdot \frac{G\cdot \varepsilon^{2/3}\cdot C^{1/2}}{700}$$

where ***ε*** is the lattice strain caused by the size difference between solute and matrix atoms, and ***C*** is the total molar ratio of the solid solution elements. In 1045 steel, the elements C, Mn, and Si can all be solved solidly in ferrite.

In addition, heterogeneous particle precipitation phases, such as carbides and silica [29,40], may exist in the 1045 steel material prepared by AFSD. The strengthening effect of the precipitated phases can be calculated by the Ashby–Orowan formula (Equation (10)).

(10) $$\sigma_{p}=\frac{0.538\cdot \mathrm{Gb}\cdot f_{v}^{1/2}}{d}\ln(\frac{d}{2b})$$

where ***f_v_*** is the volume fraction (%) of the precipitated phase and ***d*** is the diameter of the precipitated phase.

In summary, the combination of multiple toughening and strengthening mechanisms has resulted in a good balance of strength and plasticity in AFSD 1045 steel. The optimal mechanical properties achieved include a UTS of 1144.2 MPa, an elongation of 21.7%, and a PoSE of 21.7 GPa·%. However, the characterization of the fine structure of AFSD 1045 steel and the specific contribution ratio of each strengthening mechanism still require further investigation.

## 4. Conclusions

The AFSD provides a new alternative for medium-carbon steel additive manufacturing, and the effects of AFSD processing on the microstructures and mechanical properties of 1045 steel were investigated, alongside a qualitative discussion of the strengthening mechanisms. The main conclusions are as follows:

- The AFSD 1045 steel exhibits equiaxed grains with an average size of 1–3 μm, and high tool rotational speed (ω) and axial force (F) are more likely to cause the grain refinement. Additionally, there is a notable proportion of low-angle grain boundaries (56.8–60.7%) and deformed grains (58.1–63.6%), accompanied by dynamic recrystallization.
- Compared to other forming processes, AFSD 1045 steel demonstrates an excellent balance of strength and plasticity, with an ultimate tensile strength (UTS) of 1061.9–1172.3 MPa and elongation 8.6–19.0%. The product of strength and plasticity (PoSE) indicates an optimal AFSD process window with a deposition temperature (T) 850–900 °C and the product of rotational speed and force (PoRF) 4400–4800 rpm·kN.
- The remarkable plasticity of AFSD 1045 steel can be attributed to (i) the generation of delamination bifurcation cracks within the material to reduce the local stress concentration; (ii) the crack shielding effect of pile-up dislocations; and (iii) the deflection of crack propagation paths by precipitated phases. Furthermore, the combination of strengthening mechanisms, including grain boundary strengthening, dislocation strengthening, twin boundary strengthening, solid solution strengthening, and precipitation strengthening, attribute excellent overall mechanical properties to AFSD 1045 steel.

## Figures and Tables

**Table 1:** Chemical composition of 1045 steel powder (wt.%).

Element	C	Si	Mn	Cr	Ni	Cu	Fe
Content/%	0.460	0.230	0.720	0.170	0.180	0.08	Bal.

**Table 2:** AFSD test with different process parameters.

Samples No.	T/°C	ω/rpm	F/kN
1	700	400	9
2	800	400	9
3	900	400	9
4	800	400	12
5	800	400	6
6	800	500	9
7	800	300	9

**Table 3:** Tensile properties of the 1045 steel samples under different AFSD process parameters.

Samples No.	YS/MPa	UTS/MPa	Elongation/%	PoSE/GPa·%
1	910.4	976.7	4.4	4.3
2	942.4	1116.6	8.6	9.6
3	934.2	1172.3	11.6	13.6
4	853.1	1144.2	19.0	21.7
5	714.4	976.2	9.2	9.0
6	838.6	1061.9	15.8	16.8
7	860.8	893.9	5.0	4.5

## Data Availability

The original contributions presented in the study are included in the article, further inquiries can be directed to the corresponding author.

## Supplementary Materials

The following supporting information can be downloaded at: https://www.mdpi.com/article/10.3390/ma18061257/s1.
