# Supplementary material for: Effect of Additive Friction Stir Deposition Processing on the Microstructure and Mechanical Properties of 1045 Steel
Source: Materials (Basel). 2025 Mar 12;18(6):1257. doi: 10.3390/ma18061257 (PMC11943858; doi:10.3390/ma18061257)
Supplement: Supplementary file 1 [file materials-18-01257-s001.zip › materials-3407727-supplementary.pdf]

Supporting Information

Effect of Additive Friction Stir Deposition Processing on the Microstructure and Mechanical Properties of 1045 Steel

Wei Lei [1], Xudong Ran [1,2], Hulin Wu [1], Qi Wang [1], Yang Wu [1], Jipeng Sun [1], Feiyue Zhang [1], Shuhai Huang [1], Lin Xiang [1], Jianquan Tao [1,*] and Qiang Chen [1,*]

[1] Southwest Technology and Engineering Research Institute, Chongqing 400039, China; autismpait@gmail.com (W.L.); xudongran0828@outlook.com (X.R.); hlwusteri@163.com (H.W.); bruce_xlin@163.com; (Q.W.) cquwuyang@163.com (Y.W.); sun1554384191@163.com (J.S.); 18723003055@163.com (F.Z.); hsh82@163.com (S.H.); xlin0731@163.com (L.X.);

[2] School of Mechatronics Engineering, Harbin Institute of Technology, Harbin 150001, China;

* Correspondence: jarryallen@163.com (J.T.); 2009chenqiang@163.com (Q.C.)

Table S1. Correlation of AFSD 1045 steel sample grain size with T, ω, F, and PoRF.

Samples No.	T/℃	ω/rpm	F/kN	PoRF/ rpm·kN	Grain Size/ μm
1	700	400	9	3600	2.07
2	800	400	9	3600	2.05
3	900	400	9	3600	1.90
4	800	400	12	4800	1.82
5	800	400	6	2400	2.48
6	800	500	9	4500	1.84
7	800	300	9	2700	2.13

In Table S2, ***Mean*** represents the mean value of AFSD 1045 hardness and ***SD*** indicates the standard deviation.

Table S2. Hardness of AFSD 1045 steel-deposited sample.

Samples No.		*1*	*2*	*3*	*4*	*5*	*6*	*7*
Upper/ HV	*Mean*	*417.1*	*423.7*	*441.0*	*429.5*	*376.1*	*418.2*	*384.9*
	SD	*34.2*	*45.0*	*36.6*	*45.1*	*26.1*	*60.6*	*43.6*
Middle / HV	*Mean*	*303.1*	*328.2*	*331.6*	*318.6*	*261.0*	*307.3*	*285.2*
	SD	*34.7*	*28.8*	*26.8*	*30.2*	*43.7*	*47.3*	*30.4*
Bottom / HV	*Mean*	*251.7*	*279.0*	*297.9*	*285.1*	*216.9*	*276.4*	*262.4*
	SD	*3.1*	*19.5*	*13.5*	*8.1*	*42.5*	*33.1*	*11.5*
